# Assessing setup errors and shifting margins for planning target volume in head, neck, and breast cancer

**DOI:** 10.25122/jml-2022-0241

**Published:** 2023-03

**Authors:** Hayder Alabedi

**Affiliations:** 1 Department of Surgery, College of Medicine, Baghdad University, Baghdad, Iraq

**Keywords:** shifting margins, planning target volume, head cancer, neck cancer, breast cancer

## Abstract

Accurately calculating setup errors is crucial in ensuring quality assurance for patients undergoing radiation therapy treatment. This cross-sectional study aimed to determine the systematic, random, and planning target volume (PTV) margin errors for patients with head and neck cancer (n=48) and breast cancer (n=50). The treatment setup was performed using electronic portal imaging (EPIDs) and irradiated using Elekta linac. The errors were calculated using the van Herk formula. The systematic error for the head and neck was 0.89, 0.43, and 1.49 mm on the x, y, and z-axis, respectively, and 0.39, 0.74, 0.38 for the breast cases. The random error was 0.82, 0.68, 0.94 mm for the head and neck and 0.66, 0.72, 0.79 mm for the breast. The PTV margin shifting error for the head and neck were 2.79, 1.55, and 4.38 mm, while it was 1.43, 2.35, and 1.50 mm for the breast. The setup errors varied according to the tumor location. The study highlights the potential benefits of using EPIDs for reducing uncertainties in setup verification procedures.

## INTRODUCTION

Cancer is a disease that results from the abnormal growth of cells due to genetic mutations in DNA. Radiation therapy is a standard treatment for cancer that involves delivering high-energy radiation to the tumor to kill cancer cells. Accurate patient positioning before each fraction is crucial in radiotherapy treatment to ensure that the maximum dose reaches the planning target volume (PTV) while minimizing radiation exposure to organs at risk (OAR), which is the primary goal of radiotherapy [1-4]. The critical organs close to the head and neck tumors, such as optic nerves, brain stem, and cochlea, often necessitate stringent PTV margins [5]. The clinical target volume (CTV) is multiplied by a margin to account for PTV imperfections in patient positioning, beam alignment, and organ movements (i.e., setup margin and internal margin). Setup margins are important to avoid irradiating OARs inadvertently and have a significant impact on the total dose delivered to the target [4,6]. Two kinds of errors occur in radiotherapy treatment positioning: systematic error and random error. Systematic errors result in a dose distribution that deviates from the intended target area, while random errors can cause the cumulative dose to be displaced from its proper position. Systematic errors are particularly concerning since they can persist across multiple treatment sessions and potentially result in tumor recurrence or severe organ injury, while random errors tend to occur less frequently [7]. Electronic portal imaging devices (EPIDs) are often used in 3D conformal radiotherapy planning to improve the accuracy of target localization and patient positioning [8-10] and are considered effective tools for reducing and evaluating setup errors [11,12]. This study aimed to evaluate the systematic and random errors in patient positioning by calculating the shifts in the planning target volume (PTV) margins for patients with head and neck or breast cancer.

## MATERIAL AND METHODS

### Patient selection

This cross-sectional study was conducted at Medical City in Baghdad, Iraq, and included 48 patients with head and neck cancer and 50 with unilateral breast cancer. All patients were diagnosed by an oncologist and radiologist.

### Computed Tomography (CT) simulation and planning

The patients underwent computed tomography simulation (Philips, Netherlands) to obtain a 3D anatomical image of the treated site. For head and neck patients, a 5-point thermoplastic mask was used for immobilization, and the CT simulation was performed one week prior to the first radiotherapy fraction. The patients were positioned in a supine position with their heads facing forward during the scan. The thermal guide layer was embedded in thermoplastic material containing radio-opaque markers for accurate patient positioning and target localization during CT simulation and planning. The 3D anatomical images obtained from the computed tomography simulation had a slice thickness of 3 mm. These images were imported into the Monaco v5.1 treatment planning system software (Elekta Medical System, Stockholm, Sweden) for contouring using the 3DCRT technique. The radiation oncologist delineated the tumor, also known as the gross target volume-clinical target volume (GTV-CTV), along with the organs at risk surrounding the tumor in order to optimize the radiation dose to the target and minimize exposure to surrounding healthy tissue, thus defining the planning target volume (PTV). For head and neck (H&N) plans, PTV was generated with an isotropic margin of 7 mm added around the defined CTV. The prescribed dose was delivered to the patient using 6 MV and 10 MV photon beam energies with a Synergy linear accelerator (Elekta Medical System, Stockholm, Sweden).

### Electronic Portal Imaging (EPI)

Before each therapy session, patients were immobilized utilizing appropriate positioning devices, and their position was confirmed through laser alignment or skin/mask markings within the treatment room. Orthogonal portal images were obtained using a high-resolution, flat-panel, amorphous silicon digital portal imaging system with a 1024 x 768 pixels resolution. These images were compared to digitally reconstructed radiographs (DRRs) generated from orthogonal portal images obtained at 0° (anterior) and 90° (lateral) (TPS) using treatment planning software. Three translational axes (vertical (Y), longitudinal (Z), and lateral (X) were employed to study patient setup issues.

### Error analysis

To compute systematic and random errors, translational displacement was measured in three directions. Systematic error (Ʃ) for H&N and breast cases were calculated when the planned patient position differed from the individual patient positions by the standard deviation (SD) between the planned patient position and the individual patient positions for each treatment fraction, or the SD of all individual means for each direction. Random errors (σ) were defined as deviations between different treatment fractions taken weekly during the treatment. It can be determined by calculating the mean root square of the individual SD of all patients [13]. Furthermore, we estimated the value of 3D vector lengths and measured their size. In order to quantify the systematic error, the standard deviation of the average value of the individual mean setup error for each horizontal, longitudinal, and lateral direction was used (Ʃ). The random error (σ) was estimated by calculating the mean root square of the individual standard deviations along the vertical, longitudinal, and lateral axes [14]. The PTV margins were calculated using the van Herk formula [15] as follows:

PTV margin = 2.5 ∑ + 0.7 σ

The van Herk formula provides an analytical description of the effect of random and systematic geometrical deviations on the target dose to derive margin rules.

## RESULTS

The characteristics of the head and neck (H&N) and breast cancer patients included in this study are provided in Tables 1 and 2.

**Table 1:** Characteristics of head and neck patients.

**Gender**	
**Male**	34 (70.8%)
Female	14 (29.2%)
Age (years)	49.4 (35–79)
**Stage**	
I	14
II	10
III	16
IV	8
**Chemotherapy**	
Treated	36
Not treated	12

**Table 2:** Characteristics of patients with breast cancer.

**Gender**	
Male	1 (2%)
Female	49 (98%)
Age (years)	46.1 (32–73)
**Stage**	
I	17
II	21
III	7
IV	5
**Chemotherapy**	
Treated	42
Not treated	8

Orthogonal image pairs were acquired using electronic portal imaging (EPIs) for each patient, with a total of 288 image pairs for head and neck cases and 300 image pairs for breast cases. These images were then measured and corrected for systematic and random errors, as illustrated in Table 3.

**Table 3:** Characteristics of head and neck patients.

Treatment site	Head and neck			Breast		
Direction	VRT	LAT	LONG	VRT	LAT	LONG
Systematic Error (mm)	0.89	0.43	1.49	0.39	0.74	0.38
Random Error (mm)	0.82	0.68	0.94	0.66	0.72	0.79

**Figure 1:**
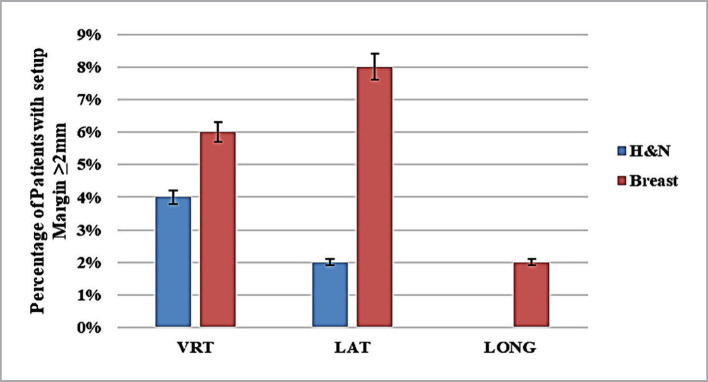
Percentage of patients who exceeded the setup error limit (≥2 mm).

The results indicate that the overall systematic error was higher than the random errors for both treatment sites (head and neck and breast), except for the lateral direction, where the random error was higher than the systematic error. The systematic and random errors for H&N subjects were greater than those of breast cases in the vertical and longitudinal (and opposite) directions and lower in the lateral (and opposite) directions. The lower error was systematically found in the longitudinal direction for the breast cases followed by the vertical direction. The accepted threshold for setup error in our center was equal to or higher than 2 mm for both studied sites. The percentage of patients with errors above the tolerance is shown in Figure 1. Only 4% of the patients had movements exceeding 2 mm in all three study directions, according to the International Electrotechnical Commission (IEC). About 2% of patients showed motion above 2 mm in two of the three study directions, while no motion was observed in the third [16]. For H&N patients, 4%, 2%, and 0% of the studied cases showed movements exceeding 2 mm in the vertical, lateral, and longitudinal directions, respectively. In contrast, the percentage was higher for breast cancer patients, with 6%, 8%, and 2% of cases showing movements exceeding 2 mm in the vertical, lateral, and longitudinal directions, respectively.

The van Herk equation was used to calculate the planning target volume (PTV), and the results are presented in Table 4. The vertical axis represents the anteroposterior (AP) direction, the lateral direction is the mediolateral (ML) direction, and the longitudinal represents the superior-inferior (SI) direction. In head and neck cases, the greatest shifting in PTV margins was found in the longitudinal direction followed by vertical and lateral directions. Conversely, in breast cases, the greatest shifting was found in the lateral direction, followed by vertical and longitudinal directions. The magnitude of the margin shifts was lower in breast cancer cases than in H&N cancer cases in the vertical and longitudinal directions but higher in the lateral direction.

**Table 4:** Shifted planning target volume (PTV) margins calculated in mm for H&N and breast cases across different directions.

Direction	Head and neck	Breast
Vertical (AP)	2.79	1.43
Lateral (ML)	1.55	2.35
Longitudinal (SI)	4.38	1.50

## DISCUSSION

Shifting errors in H&N and breast cancer cases are common setup errors in radiotherapy centers. EPIDs can assess patient positioning for 3DCRT plans and detect and correct isocenter errors during treatment to increase accuracy. In this study, EPIs were used to assess inter-fraction positioning errors for H&N and breast cancer cases in the vertical (AP), lateral (ML), and longitudinal (SI) directions. Furthermore, PTV margins were calculated using van Herk’s formula [13].

Our results showed that H&N cases had more systematic and random errors compared to breast cases, but a lower percentage of patients had errors equal to or higher than 2 mm. This is likely due to the rigorous geometry of the breast setup, with minor day-to-day alterations. These results agreed with Murthy et al. [17], who also found that breast cancer cases had a higher frequency of errors than H&N.

The setup uncertainties in H&N and breast cases can be caused by several factors, including the enlargement of the tumor area due to swelling after irradiation, changes in patient weight during fractions that can alter the delineation of the tumor, and the snug fit of the thermoplastic mask on the head and organs [18-20]. To address these situations, our center has implemented a solution by changing the immobilization degree for patients after obtaining a new CT image and delineation. However, for breast cases, setup errors can still occur due to the lung movement during CT simulation imaging or portal imaging, which can cause skin markings to shift into the treatment areas due to optical illusion and inaccurate laser and patient body alignment [21-23].

Our study identified that H&N cases had higher systematic errors than random errors in two directions, whereas breast cases had the opposite pattern. A previous study by Hurkmans et al. found that in typical clinical practice, both systematic and random errors may be less than 2 mm. [24]. The variation in the treated sites results may depend on the anatomical nature and patient immobilization technique. Our results disagreed with another study [25], which investigated 25 individuals with H&N lesions and utilized different isocenter infractions and frequent online verification to reduce systematic errors. Increasing the frequency of online verification has been proposed to reduce setup errors for PTV [26]. However, studies reported that intrafraction uncertainty and variations in organ delineation could increase errors. The accuracy of patient positioning during radiotherapy treatment can be influenced by several factors, including the optical illusion and alignment inaccuracies of lasers and lines on the patient's body. Other potential factors include equipment failure, illness, medication, and the positioning of the scanner. In general, adequate time was allocated to properly position patients for treatment. However, setup errors observed at the start of treatment may be attributed to inadequate patient comfort and/or positioning during the masking procedure, resulting in a less effective setup. Proper placement, such as pushing the shoulders caudally, closing the patient's mouth, extending the neck, and applying a tattoo on the chest, can minimize errors during simulation [27,28]. Implementing daily guidelines and fixed correction protocols can also be effective in minimizing errors.

## CONCLUSION

The present study is the first of its kind in our centers in Baghdad, providing valuable insights into the systematic and random errors associated with patient setup and calculating the corresponding PTV margin shifts. Our findings reveal that the setup errors and uncertainties vary with tumor location and can be reduced through image-guided verification. The use of image guiding, particularly EPIDs, can improve patient positioning accuracy and reduce PTV margins, which can lead to a decrease in the risk of radiation-induced complications.

## References

[1] Alwakeel AF, Al Musawi MS, Alabedi HH, Mohammed HJ (2021). Diametric assessment of IMRT treatment planning for unilateral breast cancer patient using Octavius phantom detector. Applied Nanoscience (Switzerland).

[2] Faraj MK, Naji NA, Alazawy NM (2018). The efficiency of the prescribed dose of the gamma knife for the treatment of trigeminal neuralgia. Interdiscip Neurosurg.

[3] Jubbier ON, Abdullah SS, Alabedi HH, Alazawy NM, Al-Musawi MJ (2021). The Effect of Modulation Complexity Score (MCS) on the IMRT Treatment Planning Delivery Accuracy. Journal of Physics: Conference Series; Bristol.

[4] Abdulbaqi AM, Abdullah SS, Alabed HH, Alazawy NM (2020). The Correlation of Total MU Number and Percentage Dosimetric Error in Step and Shoot IMRT with Gamma Passing Rate Using OCTAVIUS 4D-1500 Detector Phantom. Ann Trop Med Public Health.

[5] Anjanappa M, Rafi M, Bhasi S, Kumar R (2017). Setup uncertainties and PTV margins at different anatomical levels in intensity modulated radiotherapy for nasopharyngeal cancer. Rep Pract Oncol Radiother.

[6] Menzel HG (2010). The international commission on radiation units and measurements. Journal of the ICRU.

[7] Van Herk M (2004). Errors and Margins in Radiotherapy. Semin Radiat Oncol.

[8] Oh SA, Yea JW, Kang MK, Park JW, Kim SK (2016). Analysis of the setup uncertainty and margin of the daily ExacTrac 6D image guide system for patients with brain tumors. PLoS One.

[9] Mesías MC, Boda-Heggemann J, Thoelking J, Lohr F (2016). Quantification and assessment of intrafraction setup errors based on cone beam CT and determination of safety margins for radiotherapy. PLoS One.

[10] Mahdavi SR, Jazayeri Gharehbagh E, Mofid B, Jafari AH, Nikoofar AR (2017). Accuracy of the dose delivery in prostate cancer patients-using an electronic portal imaging device (EPID). International Journal of Radiation Research.

[11] Langmack KA (2001). Portal imaging. British Journal of Radiology.

[12] Herman MG (2005). Clinical use of electronic portal imaging. Semin Radiat Oncol.

[13] Ghaffari H (2018). Evaluation of Patient setup Accuracy and Determination of Optimal Setup Margin for External Beam Radiation therapy using Electronic Portal Imaging Device. Cancer Ther Oncol Int J.

[14] Van Herk M, Remeijer P, Rasch C, Lebesque J V (2000). The probability of correct target dosage: Dose-population histograms for deriving treatment margins in radiotherapy. Int J Radiat Oncol Biol Phys.

[15] Killoran JH, Kooy HM, Gladstone DJ, Welte FJ, Beard CJ (1997). A numerical simulation of organ motion and daily setup uncertainties: implications for radiation therapy. Int J Radiat Oncol Biol Phys.

[16] Murthy K, Al-Rahbi Z, Sivakumar S, Davis C (2008). Verification of setup errors in external beam radiation therapy using electronic portal imaging. J Med Phys.

[17] Schubert LK, Westerly DC, Tomé WA, Mehta MP (2009). A Comprehensive Assessment by Tumor Site of Patient Setup Using Daily MVCT Imaging From More Than 3,800 Helical Tomotherapy Treatments. Int J Radiat Oncol Biol Phys.

[18] Den RB, Doemer A, Kubicek G, Bednarz G (2010). Daily Image Guidance With Cone-Beam Computed Tomography for Head-and-Neck Cancer Intensity-Modulated Radiotherapy: A Prospective Study. Int J Radiat Oncol Biol Phys.

[19] Li H, Zhu XR, Zhang L, Dong L (2008). Comparison of 2D Radiographic Images and 3D Cone Beam Computed Tomography for Positioning Head-and-Neck Radiotherapy Patients. Int J Radiat Oncol Biol Phys.

[20] Oh YK, Baek JG, Kim OB, Kim JH (2014). Assessment of setup uncertainties for various tumor sites when using daily CBCT for more than 2200 VMAT treatments. J Appl Clin Med Phys.

[21] Delishaj D, Ursino S, Pasqualetti F, Matteucci F (2018). Set-up errors in head and neck cancer treated with IMRT technique assessed by cone-beam computed tomography: A feasible protocol. Radiat Oncol J.

[22] van der Heide UA, Kotte ANTJ, Dehnad H, Hofman P (2007). Analysis of fiducially marker-based position verification in the external beam radiotherapy of patients with prostate cancer. Radiother Oncol.

[23] Madlool SA, Abdullah SS, Alabedi HH, Alazawy N (2020). Optimum Treatment Planning Technique Evaluation For Synchronous Bilateral Breast Cancer With Left Side Supraclavicular Lymph Nodes. Iranian Journal of Medical Physics.

[24] Hurkmans CW, Remeijer P, Lebesque J V, Mijnheer BJ (2001). Set-up verification using portal imaging; review of current clinical practice. Radiother Oncol.

[25] Gupta T, Chopra S, Kadam A, Agarwal J (2007). Assessment of three-dimensional set-up errors in conventional head and neck radiotherapy using electronic portal imaging device. Radiat Oncol.

[26] Rudat V, Hammoud M, Pillay Y, Alaradi AA (2011). Impact of the frequency of online verifications on the patient set-up accuracy and set-up margins. Radiat Oncol.

[27] Pehlivan B, Pichenot C, Castaing M, Auperin A (2009). Interfractional set-up errors evaluation by daily electronic portal imaging of IMRT in head and neck cancer patients. Acta Oncol.

[28] Kim SH, Oh SA, Yea JW, Park JW (2019). Prospective assessment of inter-or intra-fractional variation according to body weight or volume change in patients with head and neck cancer undergoing radiotherapy. PLoS One.

